# Responses to flooding of plant water relations and leaf gas exchange in tropical tolerant trees of a black-water wetland

**DOI:** 10.3389/fpls.2013.00106

**Published:** 2013-05-01

**Authors:** A. Herrera

**Affiliations:** Centro de Botánica Tropical, Instituto de Biología Experimental, Universidad Central de VenezuelaCaracas, Venezuela

**Keywords:** flooding, photosynthesis, sap flux, stomatal conductance, tolerance

## Abstract

This review summarizes the research on physiological responses to flooding of trees in the seasonal black-water wetland of the Mapire River in Venezuela. Inter-annual variability was found during 8 years of sampling, in spite of which a general picture emerged of increased stomatal conductance (g_s_) and photosynthetic rate (P_N_) during the flooded period to values as high as or higher than in plants in drained wet soil. Models explaining the initial inhibitory responses and the acclimation to flooding are proposed. In the inhibitory phase of flooding, hypoxia generated by flooding causes a decrease in root water absorption and stomatal closure. An increase with flooding in xylem water potential (ψ) suggests that flooding does not cause water deficit. The P_N_ decreases due to changes in relative stomatal and non-stomatal limitations to photosynthesis; an increase in the latter is due to reduced chlorophyll and total soluble protein content. Total non-structural carbohydrates (TNC) accumulate in leaves but their content begins to decrease during the acclimatized phase at full flooding, coinciding with the resumption of high g_s_ and P_N_. The reversal of the diminution in g_s_ is associated, in some but not all species, to the growth of adventitious roots. The occurrence of morpho-anatomical and biochemical adaptations which improve oxygen supply would cause the acclimation, including increased water absorption by the roots, increased rubisco and chlorophyll contents and ultimately increased P_N_. Therefore, trees would perform as if flooding did not signify a stress to their physiology.

## Introduction

Flooding brings about a number of changes to the soil environment, including among others reduction in oxygen concentration (Kozlowski, 1984), generation of reduced ions and, additionally, diminution of irradiance incident on leaves. Flood tolerance depends on the depth of the water column, the duration of the flooded period and specific plant traits (Colmer and Voesenek, 2009). Partial or total oxygen deficiency in the roots of higher plants causes morphological, anatomical and biochemical changes, and anoxia-tolerance seems to be strictly organ-specific (Drew, 1997); such changes allow plants of tolerant species to revert to normoxic condition as flooding progresses (Pezeshki, 1993).

A large number of articles have been published on physiological responses to waterlogging/flooding in temperate tolerant plants, both herbs and trees, as well as the tropical herb, rice, but less is known about the physiological, let alone biochemical or molecular, responses of tropical species, especially trees. I here review the existing literature on tropical tolerant trees, while comparing these with temperate tolerant and intolerant species.

Flood tolerance varies greatly with, among other factors, plant species and age, and time and duration of flooding (Kozlowski, 1997). A reduction in g_s_ and P_N_ are the earliest response to flooding observed in intolerant as well as tolerant species (Pezeshki, 1993; Batzli and Dawson, 1997; Fernández et al., 1999). In tropical flood-tolerant trees, reductions in P_N_ and g_s_ are reverted later on as flooding progresses (Fernández et al., 1999; Rengifo et al., 2005), as shown previously in temperate flood-tolerant trees (Pezeshki, 1993).

One of the main reasons why hypoxia due to flooding reduces g_s_ is decreased root water absorption (Kozlowski, 1984; Tournaire-Roux et al., 2003) through reductions in root hydraulic conductivity (Aroca et al., 2011). The decrease in P_N_ observed in flood-tolerant herbaceous and tree species is apparently governed not only by stomatal but also by non-stomatal (mesophyll) factors (Pezeshki, 1987, 1993).

In riparian Panamazonian forests, trees can suffer regular, long-lasting and deep flooding, when white-water rivers such as the Orinoco or black-water rivers such as the Rio Negro overflow, thus creating floodplain ecosystems called in the first case várzea and in the second, igapó (Prance, 1979). These ecosystems differ, among other traits, in quality of the waters; várzeas have nutrient-rich, turbid and higher pH waters, whereas in the igapós waters are very nutrient-poor, transparent and acidic. These characteristics are bound to affect plant physiology and growth differently. Amazonian wetlands cover over 1,000,000 km^2^ (Melack et al., 2004), more than three times the area of the British Isles; therefore, knowing how tropical plants cope with flooding becomes of global importance.

Flood tolerance has been extensively examined in herbaceous or small floating and submerged angiosperms (revised by Colmer and Voesenek, 2009). Here, I will be dealing with large trees, which will surely make a difference because of resistances encountered by long-distance transport of O_2_ and the slow diffusion of this gas in water. In tropical wetlands, the water-column may be as high as 10–15 m and the flood period last up to 7 months (Vegas-Vilarrúbia and Herrera, 1993a; Parolin et al., 2006).

**Figure 1:**
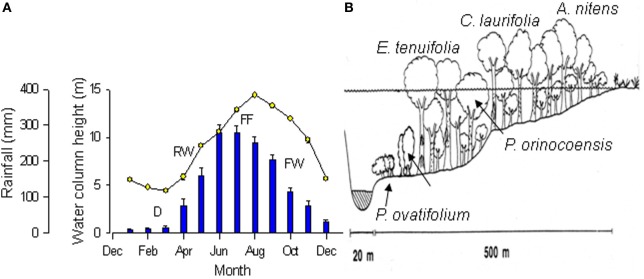
**(A)** Changes over the year in rainfall (bars) and water column height (circles) of the Mapire River relative to the Orinoco. Values are mean ± *SE* (*n* = 4 years). Stages of the flooding cycle are indicated as drainage (D), rising-waters (RW), full flooding (FF), and falling-waters (FW). **(B)** Schematic view of the vegetation and water level along a gradient from the Mapire bed to the savanna. Approximate location of species indicated. Modified from Fernández et al. (1999) and Herrera et al. (2008b).

**Figure 2:**
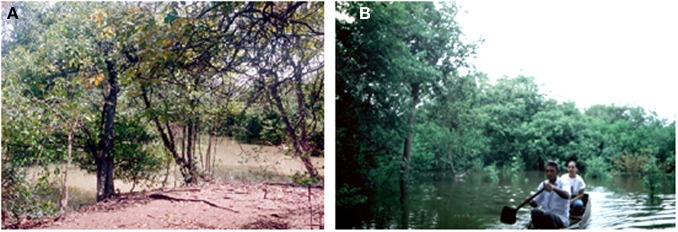
**The Mapire igapó forest during (A) drainage and (B) under full flood**.

This review summarizes the results of research on physiological responses to flooding of trees in the seasonal igapó of the Mapire River. This igapó is formed when the black-water Mapire River, a northern tributary of the Orinoco, increases its flow due to rainfall and is additionally dammed by the Orinoco. Four broad phases can be defined in the flood cycle (Figure 1A): drainage (D, December–March), rising-waters (RW, April–May), full flood (FF, June–August), and falling-waters (FW, September–November). The igapó lake covers trees at various heights depending on tree size and position along a gradient from the savanna to the river channel (Figure 1B). The species that have been examined along this gradient are *Acosmium nitens* (Papilionaceae), *Campsiandra laurifolia* (Mimosaceae), *Eschweilera tenuifolia* (Lecythidaceae), *Pouteria orinocoensis* (Sapotaceae), *Symmeria paniculata* (Polygonaceae), and *Psidium ovatifolium* (Myrtaceae). The aspect of this ecosystem at drainage and full flood is shown in Figure 2. All species in the Mapire igapó are evergreen. Several, such as *C. laurifolia*, *S. paniculata*, and *P. ovatifolium*, retain leaves underwater.

**Figure 3:**
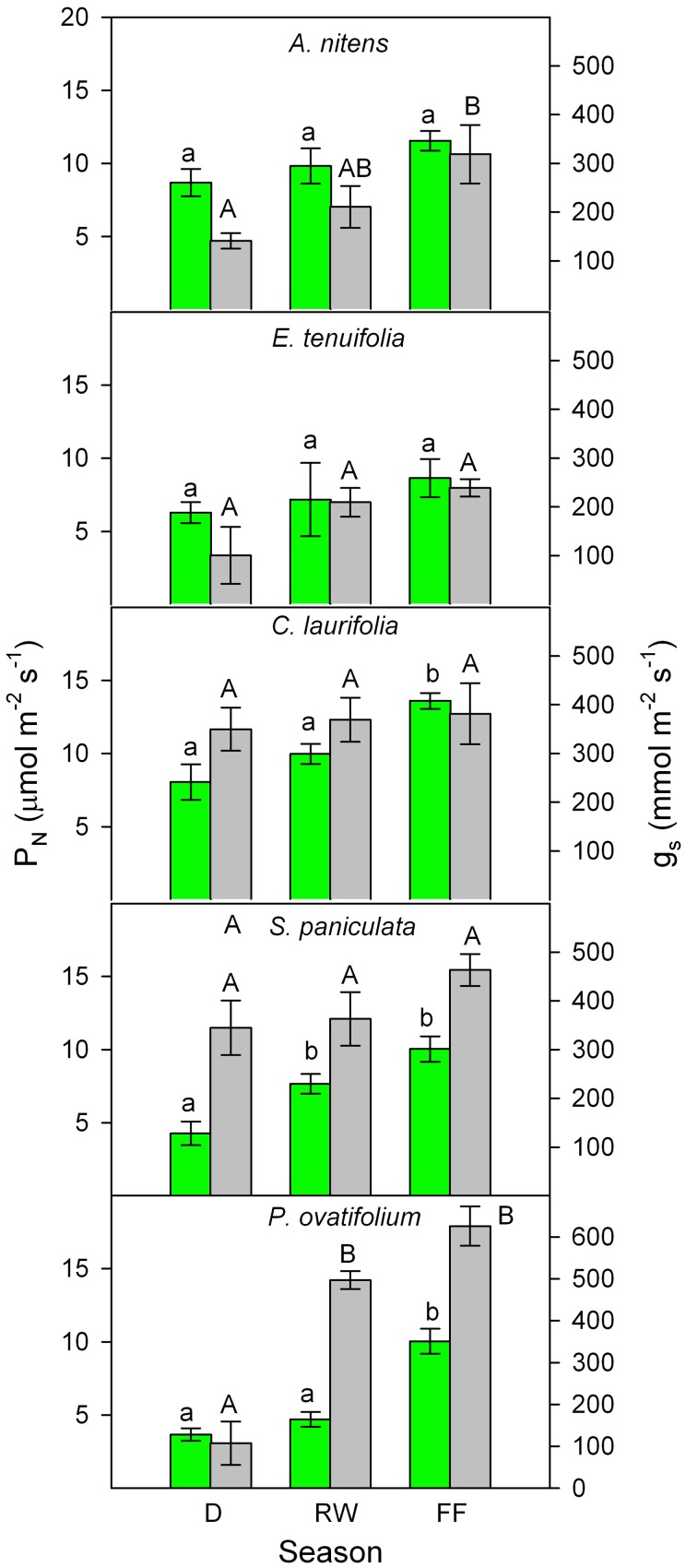
**Seasonal changes in photosynthetic rate (green) and stomatal conductance (gray) of the species indicated.** Values are mean ± *SE* of measurements were made during 1995, 1996, 1998, 1999, 2000, 2003, 2004, and 2005. Different letters indicate significant differences at *p* < 0.05. Modified from Fernández et al. (1999), Rengifo et al. (2005), and Herrera et al. (2008a).

**Figure 4:**
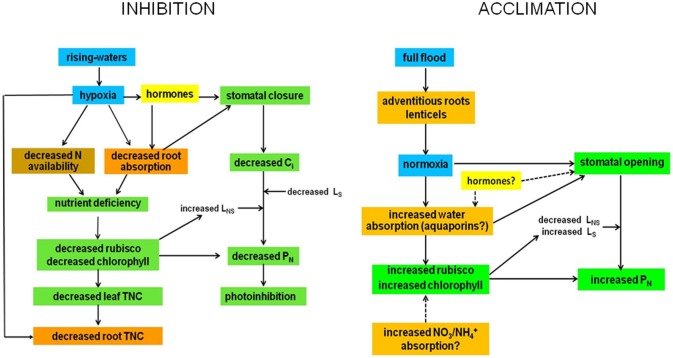
**Possible mechanisms of inhibition and acclimation of photosynthesis by flooding.** Blue, water; brown, soil; orange, roots and stems; green, leaf; yellow, undetermined.

Similar plant responses have been repeatedly observed during the various stages of the flood cycle. I will focus this review on results obtained in the field by Fernández et al. (1999), Rengifo et al. (2005), and Herrera et al. (2008a,b). In spite of inter-annual variability in gas exchange found during eight non-consecutive years of sampling, a general picture emerged of increased g_s_ and P_N_ during the flooded period to values as high as or higher than in plants in drained yet wet soil (Figure 3). The inter-annual variability may be due, among other factors, to differences in the exact timing of the flood phase, leaf developmental stage, rainfall, air temperature, and relative humidity. For example, measurements made in 1998 (Rengifo et al., 2005) were most certainly affected by the fact that this, as opposed to the other years of sampling, was a strong El Niño year. Based on these results and the literature, model are proposed (Figure 4) explaining physiological responses to the inhibitory and the acclimatized phases of flooding in species growing exclusively in this ecosystem.

I will present evidence in favor or against the proposed models. Results on water status, leaf gas exchange and Total non-structural carbohydrates (TNC) are shown in Table 1 and will be discussed where pertinent. The following issues will be examined: (1) Just how low does oxygen concentration become and how does it affect root water absorption and water status? (2) Are reductions in g_s_ and P_N_ under flooding reversible? (3) Does flooding cause photoinhibition? (4) Are changes in P_N_ under flooding caused by changes in stomatal and non-stomatal limitations? (5) How does leaf carbohydrate balance change during flooding?

**Table 1:** **Changes from drainage (D) to full-flooding (FF) in morning leaf water ( ψ) and osmotic ( ψ_**s**_) potential, maximum quantum yield of PSII (F_**v**_/F_**m**_), total chlorophyll content (Chl), photosynthetic rate (P_**N**_), stomatal conductance (g_**s**_), and total non-structural carbohydrate contents (TNC) as hexose equivalents of the species indicated**.

**Species**	**Season**	**ψ**	**ψ_s_**	***F*_*v*_*/F*_*m*_**	***Chl***	***P*_*N*_**	***g*_*s*_**	***TNC***
		**(MPa)**		**(μg cm^−2^)**		**(μ mol m^−2^ s^−1^)**	**(mmol m^−2^ s^−1^)**	**(g hexose m^−2^)**
*A. nitens*	D	−4.27	−4.13	0.79	28.9	8.1	207	9.4
	FF	−0.22	−3.50	0.79	22.6	12.3	399	14.4
*E. tenuifolia*	D	−1.53	−1.66	0.83	25.6	7.6	153	9.8
	FF	−0.18	−1.10	0.79	23.0	4.2	196	22.1
*C. laurifolia*	D	−1.07	−1.33	0.82	36.0	12.4	276	6.2
	FF	−0.47	−1.87	0.82	19.1	16.0	671	13.7
*S. paniculata*	D	−1.70	−2.02	0.81	34.0	15.3	1161	7.7
	FF	−0.28	−1.43	0.79	23.7	5.2	98	27.5
*P. ovatifolium*	D	−2.10	−2.53	0.84	24.0	13.3	269	15.4
	FF	−0.30	−1.50	0.80	22.2	5.7	109	26.6

Values are mean. Modified from Rengifo et al. (2005).

The significance of under-water photosynthesis for whole-plant C balance and the possible changes under flooding in leaf anatomy will be examined, and some eco-physiological and ecological considerations made.

## Models of physiological responses to the inhibitory and the acclimatized phases of flooding

Flooding at the inhibitory phase (RW) causes hypoxia, probably not anoxia. Hypoxia generates changes in hormone balance that cause stomatal closure and a decrease in absorption of water by roots, which may also promote stomatal closure. This brings about a decrease in intercellular CO_2_ concentration, C_i_ and P_N_. The decrease in g_s_ and P_N_ under high radiation and air evaporative demand could induce chronic photoinhibition. The decrease with flooding in soil N and absorption capacity of the roots creates a deficiency of N and possibly other nutrients, thus reducing the content and/or activity of rubisco, decreasing P_N_, increasing relative non-stomatal limitation to photosynthesis (L_NS_) and decreasing relative stomatal limitation (L_S_) [for definitions see Herrera et al. (2008a)]. The L_NS_ could also increase due to down-regulation of PSII. Root TNC content decreases due to both an increase in root anaerobic respiration and a decrease in leaf TNC content due to both decreased P_N_ and increased root demand.

During the acclimatized phase, the appearance under full flood of morpho-anatomical adaptations which improve oxygen supply would cause the acclimation. These structures would result in a situation of normoxia for the tree and cause stomatal opening and increased N and water absorption by the roots because of synthesis of new aquaporins. The increase in N uptake would cause an increase in rubisco and chlorophyll contents, and P_N_. Increased root water absorption would promote stomatal opening. This would also lead to increased P_N_. In the process of acclimation of P_N_ to full flood, L_NS_ decreases and L_S_ increases. The role of hormones in this acclimation is presumed but no evidence for it is available. After acclimation, the tree would perform as if flooding did not signify a stress to its physiology, growth or reproduction.

### Oxygen concentration under flooding and its effects on root water absorption and water status

Oxygen concentration in the water of the Mapire igapó decreases with flooding from 5 mg L^−1^ (300 μM) at the surface of the lake to 2 mg L^−1^ (120 μM) at a depth of 15 m (details in Herrera et al., 2008b). The K_m_ of cytochrome-c oxidase is 14 μM (Geigenberger, 2003); Armstrong et al. (2009) give a figure of 0.14 μM; whichever the values actually is, there is at least a ten times surplus of oxygen before tissues, especially in the roots, become anoxic. Therefore, even in the “worst” situation, plants do not seem to be exposed to severe oxygen stress. Nevertheless, without exact knowledge of internal root O_2_ concentration, describing the situation as hypoxia or anoxia is simply speculative. The decrease in the ATP/ADP ratio at decreasing O_2_ concentration is not paralleled by an increase in the NADPH/NAD^+^ ratio until O_2_ concentration reaches 1%, implying maintenance of aerobic respiration (Geigenberger, 2003).

Since O_2_ diffuses very slowly in water, the question remains: how much of that 120 μM O_2_ reaches root cells? The occurrence of anaerobic root respiration has been well documented in intolerant as well as tolerant species (Crawford, 1992). Anaerobic respiration has been reported in some tropical flood-tolerant trees (Joly and Crawford, 1982; Parolin and Wittmann, 2010). Indirect evidence for the occurrence of anaerobic metabolism in the Mapire igapó is a notable smell of alcohol near trees of *C. laurifolia* under FF which is not perceived at any other period of the flooding cycle. The sole direct evidence of alcoholic fermentation in a species from this ecosystem was provided by measurements of increased alcoholic dehydrogenase activity in seedlings of *A. nitens* grown under waterlogging (Izquierdo, 1988).

Changes in O_2_ concentration from RW to FF should not totally compromise aerobic respiration; nevertheless, a 60% decrease in O_2_ concentration could certainly be reflected in whole-plant and leaf performance, as seems to happen. This marked decrease in O_2_ concentration would be alleviated after a short period under flooding by the production of new morpho-anatomical structures and the operation of physiological and biochemical processes, such as pressurized gas transport and aquaporin synthesis.

In an ingenuous approach to the issue of aquaporin influence on root water absorption, McElrone et al. (2007) accessed through caves the fine roots of trees growing at a depth of 18 m and determined that aquaporin activity contributed up to 45% of hydraulic conductivity. The collection of fine roots and measurement of aquaporin activity in flooded trees should help understanding the acclimation of aerial responses to flooding.

The improvement of oxygen transport to the roots by pressurized gas transport, experimentally demonstrated in some temperate tree species (Grosse et al., 1992) and tropical flood-tolerant herbs (Konnerup et al., 2011), is one of the known adaptations to flooding. In saplings of five Amazonian tree species, internal aeration of the roots was improved under conditions of pressurized gas transport as shown by measurements of oxygen exchange between root and rhizosphere. Using a tracer gas, Graffmann et al. (2005) showed gas permeability of transport pathways between the stem base and the roots of these saplings and concluded that pressurized gas transport significantly contributes to internal aeration of roots.

In intolerant species, the initial reduction under flooding in g_s_ is accompanied by decreased ψ and leaf water status (Crawford, 1982; Ruiz-Sánchez et al., 1996). In trees of the Mapire igapó, ψ was found to increase with flooding (Table 1), suggesting that flooding does not cause water deficit in these trees. Weak or no relationship between g_s_ and ψ was found, except for *A. nitens* (*r*^2^ = 0.43), the one species growing along the gradient in the Mapire igapó that suffers from water deficit during the dry season. Since g_s_ diminished during the earlier stages of flooding, trees behaved as iso-hydric species. Similar increases with flooding in ψ without decreased P_N_ and, presumably, g_s_ were found in the evergreen várzea tree *P. glomerata* (Armbrüster et al., 2004). These results suggest a direct effect of flooding on gas exchange independent from water status.

The ABA content of leaves and xylem sap extracted under positive pressure increased from D to RW and FF in *C. laurifolia* and *P. orinocoensis*, together with the known decrease in g_s_, suggesting a positive relationship between ABA and stomatal closure. In fact, the regression between ABA and g_s_ was linear with *r*^2^ = 0.98 (Rengifo et al., 2006). A similar relationship was found in flooded tomato plants (Else et al., 1995). These results are subject to revision, since to obtain meaningful changes in sap ABA content, care must be taken when collecting sap, as over-pressurizing stems may bring out ABA located in roots (Jackson, 1997), thus portraying an erroneous picture of ABA influence on g_s_ during the flooding cycle. No relationship was found in flooded tomato plants between stomatal closure and inhibition of gibberellin or cytokinin export (Else et al., 2009). Possible methodological errors in determination of data by Rengifo et al. (2006) may render their interpretation erroneous; nevertheless, they open interesting research avenues in tolerant trees.

Trees under flood in the igapó showed values of leaf osmotic potential (ψ_s_) consistently lower than those of ψ (Table 1), suggesting the occurrence of osmotic adjustment (Rengifo et al., 2005); an osmotic adjustment of 0.26 MPa was shown to take place in flooded citrus seedlings (García-Sánchez et al., 2007). In four of the igapó species studied, soluble sugars contributed significantly to the decrease in ψ_s_; in *A. nitens*, the contribution was low presumably because nitrogenous compounds supplied by nitrogen fixation in this legume made a significant contribution (Fernández et al., 1999). The accumulation in leaves of osmotically active metabolites, mostly sugars, may be a mechanism whereby these trees tolerate flooding by increasing turgor potential, which allows the resumption of high g_s_ and P_N_.

When it is due to water transport from the roots to the atmosphere and not to replenishment of reservoirs, xylem sap flux may indicate the ability of roots to absorb water. In trees of *C. laurifolia*, sap flux changed seasonally; relative to the highest values measured during drainage in November, sap flux was 47% at the dry season, decreasing to 25% at rising-waters and resuming 49% under full flood [calculated from Herrera et al. (2008b)].

Since at RW dawn ψ remained high, it became apparent that the initial stages of flooding imposed a restriction to sap flux unrelated to water deficit. The decrease at RW in highest daytime sap flux was due to reduced leaf-specific hydraulic conductivity, whereas the recovery of daytime sap flux observed 1.5 months later was correlated to an increase in leaf-specific hydraulic conductivity, and attributed to acclimation.

The decrease with flooding in root hydraulic conductivity has been documented and attributed to cytoplasmic acidification due to increased CO_2_ concentration and inhibition of aquaporin activity (Aroca et al., 2011). Evidence of this has been gathered in herbs and shrubs, mainly of agronomic interest and flood-intolerant, but no data are still available on flood-tolerant trees. Early flooding in the Mapire igapó apparently inhibited water absorption by roots and this inhibition was overcome later on at a higher water column through an acclimation process possibly involving the improvement of internal aeration by adventitious roots or by synthesis of new aquaporins.

A substantial night-time flux accompanied by nocturnal stomatal aperture was found in *C. laurifolia* under FF (Herrera et al., 2008b). Similar observations have been made in plants of other species and from other environments (e.g., arid) and a role for night-time transpiration in N absorption has been suggested (Snyder et al., 2008). Why would flood-tolerant trees implement a mechanism for nutrient acquisition during the night remains to be elucidated. One possibility is that the development of positive nocturnal root pressure generated by active transport of ions into the xylem, together with open stomata, results in increased sap flux and nutrient supply to the shoot.

### Stomatal closure and reduction in P_N_ under flooding are reversible

In three out of four tropical tree species that grow on drained soils in drylands, experimentally subjected to waterlogging (Lopez and Kursar, 1999), g_s_ diminished 30% on average, one of the species, *Prioria copaifera*, showing no change. In this species P_N_ was significantly reduced after 45 days of experimental flooding but, in contrast to the other three species, resumed drained values after 90 days of flooding, the authors deeming this a flood-tolerant species.

In Table 2, values of maximum P_N_ of evergreen trees in a Brazilian várzea and the Mapire igapó are compared during the drained and flooded periods. None of the várzea species showed changes with flooding, whereas in the igapó P_N_ increased in all the species. The observation that although igapó waters are poorer and more acidic than várzea waters, average maximum P_N_ at FF was similar between wetlands merits further comparative research.

**Table 2:** **Maximum values of photosynthetic rate in evergreen trees growing in a várzea and an igapó during drainage (D) and flooding (FF)**.

**Species**	**Wetland**	***P*_*N*_(μmol m^−2^ s^−1^)**		**References**
		**D**	**FF**	
*Pouteria glomerata*	várzea	10.0 ± 1.3	10.4 ± 2.0	Parolin et al. (2006)
*Pouteria glomerata*	várzea	12.0	12.2	Armbrüster et al. (2004)
*Cecropia latiloba*	várzea	16.8 ± 1.5	15.0 ± 4.6	Parolin (2000)
*Senna reticulata*	várzea	20.0 ± 4.0	18.4 ± 4.3	
*Nectandra amazonum*	várzea	9.3 ± 2.4	7.6 ± 3.1	
**All**		**13.6**	**12.7**	
*Acosmium nitens*	igapó	8.7 ± 0.9	11.6 ± 0.7	
*Eschweilera tenuifolia*	igapó	6.3 ± 0.7	8.6 ± 1.3	
*Campsiandra laurifolia*	igapó	8.0 ± 1.2	13.6 ± 0.5	
*Symmeria paniculata*	igapó	4.3 ± 0.8	10.0 ± 0.9	
*Psidium ovatifolium*	igapó	3.7 ± 0.4	10.0 ± 0.9	
**All**		**6.2**	**10.8**	

Values are mean ± SE for species and mean for each wetland (in bold). Data for the igapó are means of value reported by Fernández et al. (1999), Rengifo et al. (2005), and Herrera et al. (2008a,b).

**Figure 5:**
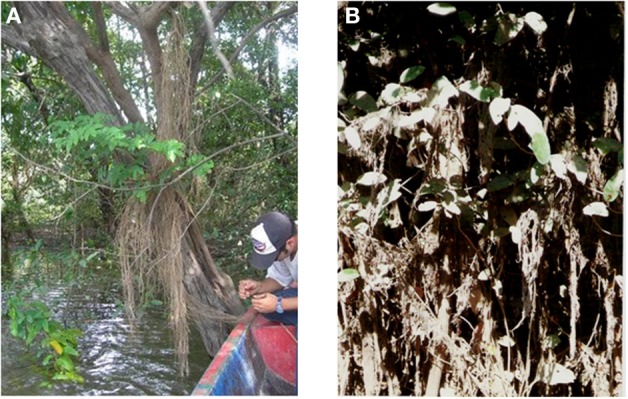
**Adventitious roots observed in October at falling-waters in trees of: (A) *Campsiandra laurifolia* and (B) *Symmeria paniculata*.** Note in **(B)** the mud deposits on leaves from previous flood.

The reversal of diminished g_s_ to values under drainage was associated in *C. laurifolia* and *S. paniculata* to the growth of adventitious roots (Figure 5). The occurrence with flooding of adventitious roots and hypertrophied lenticels has been reported in trees of several species in the central Amazonian floodplains (Parolin, 2001). Amelioration of leaf gas exchange through improvement in aeration by adventitious roots and hypertrophied lenticels has been reported in temperate tolerant species (Kozlowski, 1984) such as *L. laricina*, in which such roots increase root hydraulic conductivity apart from improving oxygenation (Islam and Macdonald, 2004).

### Flooding and photoinhibition

Despite decreased g_s_ and P_N_ and high radiation and air temperature, emerged leaves of tolerant trees do not become photoinhibited during RW, when decreased P_N_ is often observed. Values of maximum quantum yield of PSII, F_v_/F_m_ (Table 1), were always higher than 0.70, the threshold proposed by Bolhàr-Nordenkampf and Öquist (1993) for determining that photoinhibition occurs. The F_v_/F_m_ was also higher than 0.70 in plants of *S. paniculata* growing in the Solimões River várzea that had been submerged at 1 m in the dark for 6 months, and leaves submerged deeper did become photoinhibited (*F*_*v*_/*F*_*m*_ = 0.20) but regained high values several weeks later upon re-emergence (Waldhoff et al., 2002).

In the Mapire igapó, most leaves of plants of *S. paniculata* and *P. ovatifolium* under FF for approximately 6 months may become photoinhibited because the entire tree is covered with a 15-m-high water column. This, together with a reduction of 50% in radiation at a depth of 60 cm in June (Fernández et al., 1999), could mean that deeply submerged leaves do reduce P_N_ to zero. Leaves of *S. paniculata* that developed at D and remained submerged for approximately 4 months were fully functional when they emerged at FW (Fernández et al., 1999), indicating that they were not negatively affected by submergence.

### Reversible reduction in P_N_ under flooding is associated to changes in stomatal and non-stomatal limitations

It has not been systematically examined whether in flood-tolerant trees P_N_ decreases under flood due to decreased g_s_ only. In flooded plants of the tropical species *Genipa americana*, photosynthesis was co-limited by stomatal and non-stomatal factors (Mielke et al., 2003). In seedlings of the non-tolerant temperate species *Nothofagus menziesii* and *N. solandri*, P_N_, g_s_, and non-stomatal conductance, i.e., mesophyll conductance plus carboxylation efficiency, decreased markedly in response to waterlogging (Sun et al., 1995). In the igapó species *P. orinocoensis*, carboxylation efficiency in experimentally submerged seedlings decreased 70% and CO_2_-saturated P_N_ 61% relative to drained seedlings (Fernández, 2006), indicating co-limitation of photosynthesis by stomatal and non-stomatal factors.

**Figure 6:**
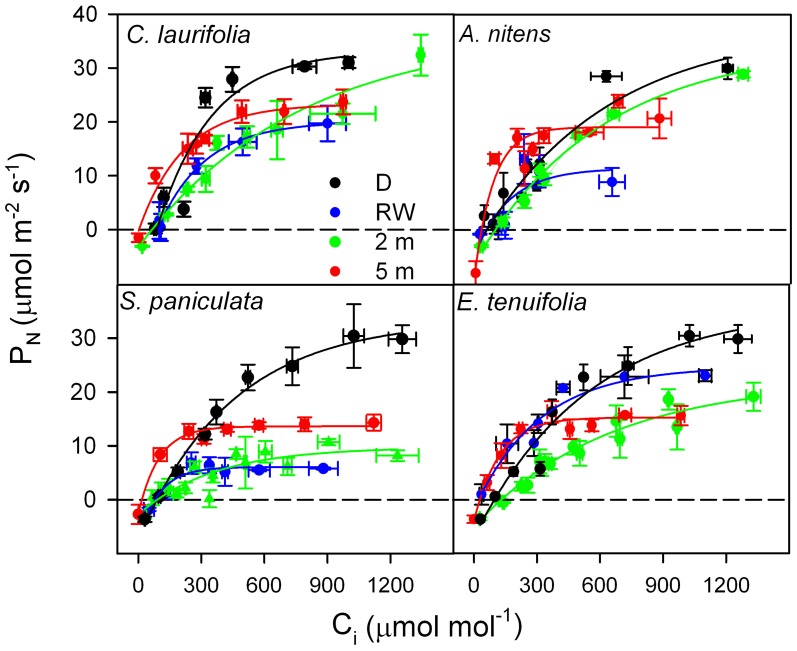
**Response curves of photosynthetic rate to intercellular [CO_2_] in the species indicated during drainage (black), rising-waters (blue), flooding by 2-m water column (green), and full flood by 5-m water-column (red).** Modified from Herrera et al. (2008a).

**Figure 7:**
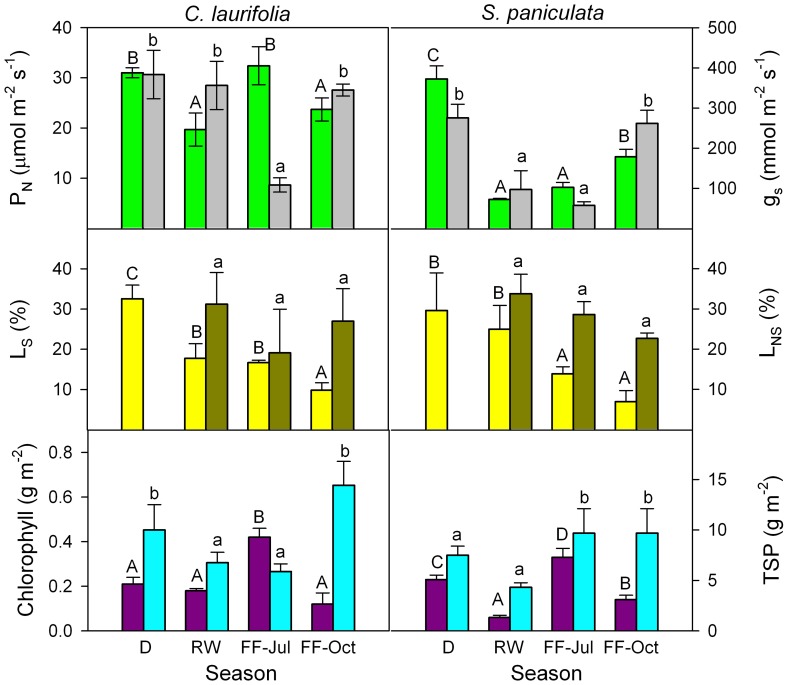
**Seasonal changes in CO_2_-saturated photosynthetic rate (green), stomatal conductance at ambient CO_2_ concentration (gray), relative stomatal (yellow) and non-stomatal (dark green) limitations of photosynthesis, chlorophyll (purple), and total soluble protein (cyan) content of the species indicated.** D, drainage; RW, rising-waters; FF-Jul, full flood in July; and FF-Oct, full flood in October. Modified from Herrera et al. (2008a).

In order to determine whether in species of the Mapire igapó P_N_ is reduced due to changes in L_S_ and L_NS_, response curves of P_N_ to C_i_ were done (Figure 6). Photosynthetic capacity was affected by flooding, as indicated by variations in CO_2_-saturated P_N_ (Figures 6, 7). The L_S_ (the difference in CO_2_-saturated P_N_ and P_N_ at ambient CO_2_ concentration divided by the former) decreased, whereas L_NS_ (CO_2_-saturated P_N_ at a given time divided by the maximum CO_2_-saturated P_N_) increased relative to drainage (Figure 7). The increase in L_NS_ was related to a decrease in total soluble protein (TSP), an indirect measure of rubisco content, and chlorophyll content (Figure 7). Nevertheless, it was previously reported that rubisco content was similar in emerged and submerged leaves of *P. ovatifolium* and higher in submerged than emerged leaves of *S. paniculata* (Fernández et al., 1999), indicating no loss of rubisco due to submergence or even flooding. It remains to determine whether this maintenance of rubisco content is a general response in the rest of the species in the igapó. A 54% change in P_N_ was due to changes in g_s_, confirming that the limitation imposed by flooding to P_N_ was not only stomatal (Herrera et al., 2008a).

Soil NO^−^_3_ content in the Mapire igapó decreases markedly during the flooded period, whereas NH^+^_4_ content progressively increases until drainage (Barrios and Herrera, 1994). The decrease in NO^−^_3_ content could be relevant for metabolism in non-leguminous but not in leguminous trees. Approximately 30% of the species in the Mapire igapó, including *A. nitens* and *C. laurifolia*, are legumes (Rosales, 1988). Setting aside differences in habit and habitat, the positive effect in the pasture legume *Lotus corniculatus* of low O_2_ concentration on nodulation, N_2_ fixation and growth reported by James and Crawford (1998) suggests that a similar response of N_2_ fixation to low O_2_ could be expected in flood-tolerant legume trees.

An increase with flood in L_NS_ in legumes would not necessarily involve a decrease in rubisco content due to low N supply, since legumes do not depend on NO^−^_3_ availability for protein synthesis. Instead, the increase in L_NS_ and decrease in TSP of legumes of the Mapire igapó could be due to low availability/absorption of other nutrients, extremely low O_2_ concentration in the nodules or toxicity caused by reduced ions. Uptake of NO^−^_3_ and NH^+^_4_ by saplings of *Fagus sylvatica* (flood-intolerant) was severely inhibited by waterlogging, whereas in *Populus tremula* × *P. alba* (flood-tolerant) absorption of both N forms was unaffected (Kreuzwieser et al., 2002). We have no information on which form of nitrogenous compounds trees, whether legumes or non-legumes, absorb at D or FF.

Leaf N content of species in the Mapire igapó (Rosales, 1988) was similar to that in the Rio Negro igapó (Parolin et al., 2002), with an average of 2% (by dry mass). No data are available for seasonal variations in leaf N content in the Mapire igapó; in the Rio Negro igapó, content varied between the drained and the flooded phases, with no consistent trend, since in some species content decreased, whereas in others it increased with flooding. Changes in content were related to leaf development, content being higher in new leaves which flushed mostly during the flooded period. Therefore, the reduction in soil N availability is not apparently reflected in leaf N content.

### Leaf carbohydrate balance changes during flooding

Information on changes in TNC content of flood-tolerant species is scant. In seedlings of the várzea species *Himatanthus sucuuba* experimentally subjected to submergence in the dark, root starch content diminished while alcoholic dehydrogenase activity remained relatively constant after an initial increase (Ferreira et al., 2009). It has been proposed that flood tolerance is proportional to the capacity of roots for TNC storage (Crawford, 1992). In the Mapire igapó, difficulty in accessing roots has impeded the determination of root TNC content; therefore, leaf TNC balance has been used as a surrogate measure.

Leaves of all the trees studied in the Mapire igapó accumulated TNC under flooding (Table 1) but this accumulation was not always related to a higher P_N_. Similarly, flooded seedlings of the temperate tolerant species *L. laricina* showed a marked increase in shoot starch after 27 days of flooding, as opposed to seedlings of the intolerant species *Picea mariana* (Islam and Macdonald, 2004), which suggests that starch accumulation in leaves is a tolerance mechanism. In our investigation, TNC accumulation began at RW, when P_N_ of all the species was lower than the maximum (Table 1). This suggests that photosynthates could not be translocated to other sinks and the mechanisms responsible for alleviating hypoxia had not begun operating. In all the species TNC content began to decrease at FF or FW, coinciding with the resumption of high P_N_ (Rengifo et al., 2005). Enhanced starch and sugar accumulation was observed in woody species under flooding, suggesting that this is a consequence of reduced phloem translocation from shoot to root (Kreuzwieser et al., 2004).

## Significance of under-water photosynthesis for whole-plant C balance

**Figure 8:**
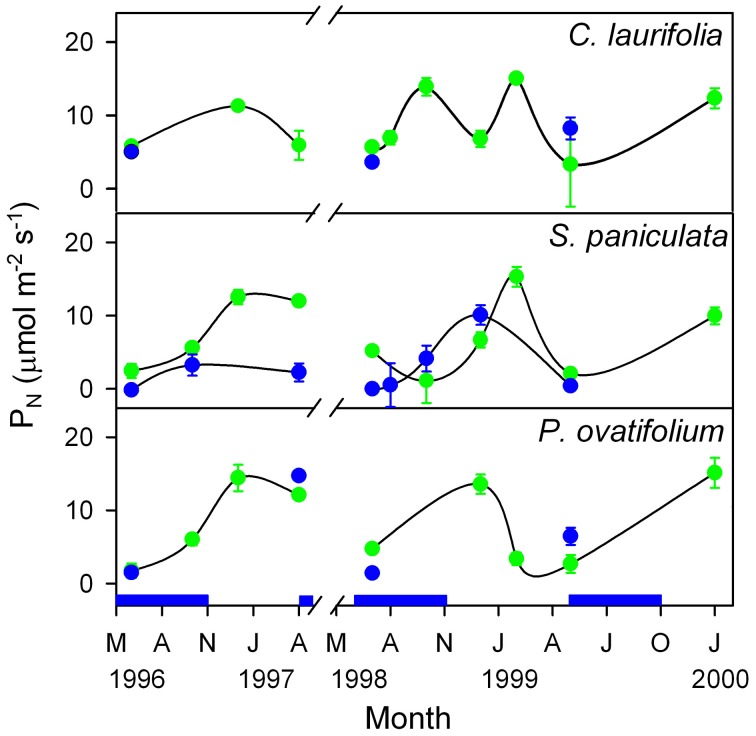
**Seasonal changes in photosynthetic rate of emerged and submerged leaves of the species indicated.** Green circles, emerged leaves; blue circles, submerged leaves. The blue indicates the duration of the flood period. Modified from Herrera et al. (2001).

In the Mapire igapó, some of the tree species maintain live leaves submerged for as long as 6 months. Submerged leaves brought to the air showed values of P_N_ similar to emerged leaves (Figure 8), indicating that leaves may be photosynthetically active under water.

The significance for whole-tree physiology of underwater photosynthesis has not been examined yet; the energy-costly process of maintaining live leaves under water may be compensated by continued O_2_ supply to the roots produced by the photosynthetic activity of these submerged leaves. Significant daily oscillations in TNC content of submerged leaves of *P. ovatifolium* and *C. laurifolia* that were 53 and 131% of those in aerial leaves, respectively (Fernández et al., 1999), support the hypothesis that these leaves are in effect photosynthesizing underwater. Additionally, the balance could resided in having leaves that are fully functional when waters fall; in this case, the payback would lie in a higher whole-tree leaf area, hence higher productivity, as opposed to trees which do not maintain live leaves under water.

The occurrence of radial oxygen loss in submerged plants has been interpreted as evidence of photosynthetic O_2_ production and transport to roots (Armstrong et al., 2006), demonstrating a positive contribution of underwater photosynthesis to whole-plant survival.

Indirect evidence of the occurrence of underwater photosynthesis is contradicted by the finding in seedlings of *P. orinocoensis* that O_2_ consumption by submerged leaves measured with an O_2_ electrode became almost zero after 12 days and remained so for 45 days, while emerged leaves, although continually reducing P_N_ and g_s_ during 45 days of treatment, had values of P_N_ after 12 days of 70% the value at the beginning of flooding (Fernández, 2006). The author argues that such responses would be expected from seedlings in the field because to them, in contrast to adult trees, flooding would be abrupt and stressful.

The case of *S. paniculata* and *P. ovatifolium* is particularly interesting, because plants of these species are covered by water earlier and remain submerged longer than other species in the igapó forest. Most of the foliage of these plants remains under water at 25% of incident radiation, yet is fully functional as soon was waters fall (Fernández et al., 1999; Rengifo et al., 2005). An important issue related to the possible underwater photosynthetic activity is that of whether enough CO_2_ is available. The CO_2_ concentration calculated at the pH and alkalinity of the water from data of Vegas-Vilarrúbia and Herrera (1993b) was at any season one order of magnitude higher than the K_m_(CO_2_) of rubisco (Fernández et al., 1999); therefore, CO_2_ availability would not be a limiting factor as long as the gas diffused into the leaf.

## Leaf anatomy remains unchanged under flooding

**Figure 9:**
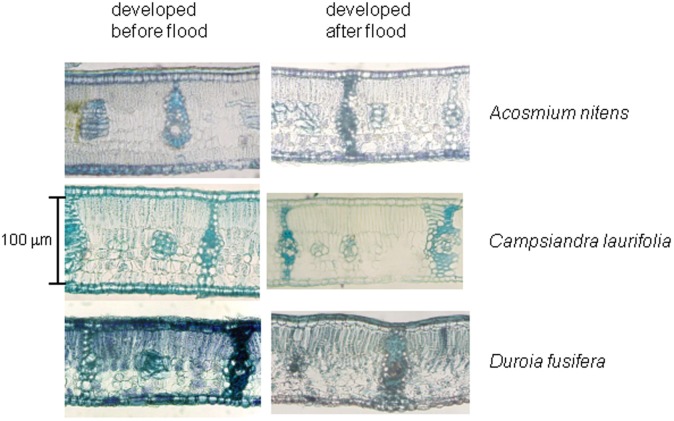
**Cross-sections of emerged leaves of the species indicated produced during drainage and after full flooding.** The sections were stained with toluidine blue. Modified from Herrera et al. (2009).

In many tolerant was well as intolerant species, flooding leads to a programmed destruction of cells in the leaves as well as stems that ends in the formation of aerenchyma, in a process designed to improve aeration of organs. In the Mapire igapó, as observed in tolerant trees of the Solimões River igapó (Waldhoff and Furch, 2002; Waldhoff, 2009), leaf anatomy remained practically unchanged regardless of phase of the flood cycle (Herrera et al., 2009; Figure 9). Emerged leaves under FF are apparently new and, using lack of sediment deposition as an indicator, produced by branches not covered by water. No signs of branch or petiole elongation under flooding, a phenomenon observed in flooded herbs (Bailey-Serres and Voesenek, 2008), have been observed but their occurrence may not be ruled out without detailed time-series on labeled individuals.

A xeromorphic leaf anatomy, such as described for drought-tolerant trees (Roth and Lindorf, 1991), was observed, with abundant sclerenchyma separating mesophyll sections into areoles, and no aerenchyma formation (Herrera et al., 2009). The significance of the occurrence of heterobaric leaves in this environment is unclear and merits further investigation.

Leaves collected in the Mapire igapó at FW from trees on drained soil and trees under flooding had few differences in proportional tissue thickness, the sole significant ones being a reduction in whole-leaf thickness of *E. tenuifolia*, *P. orinocoensis*, and *S. paniculata* and a change in relative thickness of parenchymata in *E. tenuifolia* (Herrera et al., 2009). These results contrast with observations done on the tropical species *Alchornea triplinervia* (Roças and Scarano, 2001), where palisade and spongy parenchymata of flooded plants growing in the field were 1.4 times as thin as those in unflooded plants.

Specific leaf area (SLA = area/mass) decreased with flooding, being higher in young than mature leaves and similar in mature emerged and submerged leaves (Herrera et al., 2009). In *Rumex palustris*, a wetland herbaceous plant, leaf thickness and SLA were 20 and 58% lower in submerged than emerged leaves (Mommer et al., 2005). This was attributed the function of facilitating O_2_ diffusion through the liquid phase into the mesophyll. In trees of the Mapire igapó the acclimation to flooding of leaf gas exchange may involve an increase in mesophyll conductance to CO_2_ in spite of augmented dry mass per area, which may have increased due to increased total soluble protein (Herrera et al., 2008a).

## Eco-physiological and ecological considerations

The occurrence in all the species of the Mapire igapó examined of values of g_s_ and P_N_ as high under D as under FF suggests that even though these species are indeed tolerant to flooding, they thrive under drainage as long as the soil is wet. This is supported by the finding that in *C. laurifolia*, *A. nitens*, *P. orinocoensis*, and *P. ovatifolium*, a strong relationship between the formation of new wood rings and the fluctuation of the river level during the non-flooded months suggested that an increase in the river water level during drainage promoted growth probably because of better access to ground water (Dezzeo et al., 2003). This could be interpreted as meaning that these species chose to occupy wetlands because of the competitive advantages tolerance gives them, not because of a strict requirement of flooding to grow. Trees of *A. nitens* and *C. laurifolia* grow vigorously at the savanna-end of the gradient, where they experience flooding of low height and short duration, and trees of *Andira inermis*, among others, grow to very large size in areas of the savanna far removed from the igapó. The apparent lack of need for flooding in these species contrasts with the requirement of salt in other flood-tolerant trees, mangroves, in many of which P_N_ and growth increase in response to salinity up to an optimum (Wang et al., 2011).

Flooding is frequently considered a generalized stress (e.g., Jackson and Colmer, 2005; Bailey-Serres and Voesenek, 2008; Perata et al., 2011), even when dealing with flood-tolerant plants (Parolin and Wittmann, 2010). In agreement with Otte (2001), I argue here that flooding would be stressful if it were not normally encountered by plants and that waterlogging and flooding are not stressful to wetland plants, but only to non-adapted dry land plants. The aim in this argument is not semantic; rather, I have endeavored to demonstrate that initial “negative” responses to flooding of tolerant trees are part of the adaptation. Additionally, the mere observation that ψ increases when waters rise supports the idea of not describing flooding as a stress to tolerant plants.

**Figure 10:**
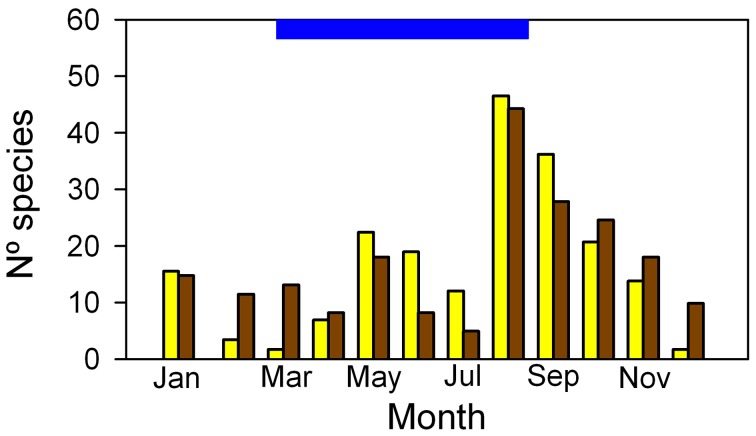
**Frequency distribution of flower (yellow) and fruit (brown) production in trees of the Mapire igapó.** The blue bar on the ordinate indicates the length of the flood period. Modified from Rosales (1988).

Of the 85 species identified in the Mapire igapó, 85% flowered and 73% set fruit during the flooded period, mostly during August-September (Figure 10). Detailed time-series of leaf production and reproduction in conspecific trees growing under flood and in dry lands would be welcome when dealing with flood tolerance. Together with high P_N_ values and maintenance of leaf area, the reproductive phenological behavior seems to indicate that flooding increases fitness through its two components, survival and fecundity. This supports the idea that flooding, far from constituting a stress to these wetland plants, is a natural and necessary part of the suite of environmental variables that make their presence in this ecosystem possible.

## Concluding remarks and future perspectives

Thus far, a wealth of information is available on the responses to flood of leaf gas exchange and leaf and whole-plant water status but we know very little about mechanisms underlying such responses. The following is important to solve this issue:

1. In order to gain knowledge on plant tolerance to hypoxia/anoxia in tropical tolerant trees, the morpho-anatomical, biochemical and molecular characteristics of roots must be literally unearthed. *De novo* synthesis of root aquaporins and the occurrence of pressurized O_2_ transport could help explain acclimation to flooding. Difficulty in accessing the root system of large trees has hindered progress in this area and data from more feasible experiments with seedlings may not necessarily be extrapolated to adult trees.
2. Examination of the hormonal balance of these trees under flooding seems imperative; preliminary data on leaf and xylem ABA contents point in the direction of promising research.
3. Long-term phenological observations under continually drained conditions should help elucidate whether these species are dependent on flooding for the completion of their life cycle.

### Conflict of interest statement

The author declares that the research was conducted in the absence of any commercial or financial relationships that could be construed as a potential conflict of interest.

## References

[B1] Armbrüster N. Müller E. Parolin P. (2004). Contrasting responses of two amazonian floodplain trees to hydrological changes. Ecotropica 10, 73–84

[B2] Armstrong J. Jones R. E. Armstrong W. (2006). Rhizome phyllosphere oxygenation in *Phragmites* and other species in relation to redox potential, convective gas flow, submergence and aeration pathways. New Phytol. 172, 719–731 10.1111/j.1469-8137.2006.01878.x 17096797

[B3] Armstrong W. Webb T. Darwent M. Beckett P. M. (2009). Measuring and interpreting respiratory critical oxygen pressures in roots. Ann. Bot. 103, 281–293 10.1093/aob/mcn177 18819952 PMC2707311

[B4] Aroca R. Porcel R. Ruiz-Lozano J. M. (2011). Regulation of root water uptake under abiotic stress conditions. J. Exp. Bot. 63, 43–57 10.1093/jxb/err266 21914658

[B5] Bailey-Serres J. Voesenek L. A. C. J. (2008). Flooding stress: acclimations and genetic diversity. Annu. Rev. Plant Biol. 59, 313–339 10.1146/annurev.arplant.59.032607.092752 18444902

[B6] Barrios E. Herrera R. (1994). Nitrogen cycling in a Venezuelan tropical seasonally flooded forest: soil nitrogen mineralization and nitrification. J. Trop. Ecol. 10, 399–416

[B7] Batzli J. M. Dawson J. O. (1997). Physiological and morphological responses of red alder and sitka alder to flooding. Physiol. Plant. 99, 653–663

[B8] Bolhàr-Nordenkampf H. R. Öquist G. (1993). Chlorophyll fluorescence as a tool in photosynthesis research, in Photosynthesis and Production in a Changing Environment, eds Hall D. O. Scurlock J. M. O. Bolhàr-Nordenkampf H. R. Leegood R. C. Long S. P. (London: Chapman and Hall), 193–206

[B9] Colmer T. D. Voesenek L. A. C. J. (2009). Flooding tolerance: suites of plant traits in variable environments. Funct. Plant Biol. 36, 665–681 10.1071/FP09144 32688679

[B10] Crawford M. M. (1992). Oxygen availability as an ecological limit to plant distribution. Adv. Ecol. Res. 23, 93–185

[B11] Crawford R. M. M. (1982). Physiological responses to flooding, in Encyclopedia of Plant Physiology, eds Pirson A. Zimmermann M. H. (Berlin: Springer-Verlag), 453–477

[B12] Dezzeo N. Worbes M. Ishii I. Herrera R. (2003). Annual tree rings revealed by radiocarbon dating in seasonally flooded forest of the Mapire River, a tributary of the lower Orinoco River, Venezuela. Plant Ecol. 168, 165–175

[B13] Drew M. C. (1997). Oxygen deficiency and root metabolism: injury and acclimation under hypoxia and anoxia. Annu. Rev. Plant Physiol. Plant Mol. Biol. 48, 223–250 10.1146/annurev.arplant.48.1.223 15012263

[B14] Else M. A. Davies W. J. Malone M. Jackson M. B. (1995). A negative hydraulic message from oxygen-deficient roots of tomato plants? Plant Physiol. 109, 1017–1024 10.1104/pp.109.3.1017 12228649 PMC161404

[B15] Else M. A. Janowiak F. Atkinson C. J. Jackson M. B. (2009). Root signals and stomatal closure in relation to photosynthesis, chlorophyll a fluorescence and adventitious rooting of flooded tomato plants. Ann. Bot. 103, 359–376 10.1093/aob/mcn208 19001430 PMC2707317

[B16] Fernández M. D. (2006). Changes in photosynthesis and fluorescence in response to flooding in emerged and submerged leaves of *Pouteria orinocoensis*. Photosynthetica 44, 32–38

[B17] Fernández M. D. Pieters A. Donoso C. Herrera C. Tezara W. Rengifo E. (1999). Seasonal changes in photosynthesis of trees in the flooded forest of the Mapire river. Tree Physiol. 19, 79–85 10.1093/treephys/19.2.79 12651586

[B18] Ferreira C. S. Piedade M. T. F. Franco A. C. Carvalho Goncalves J. F. Junk W. F. (2009). Adaptive strategies to tolerate prolonged flooding in seedlings of floodplain and upland populations of *Himatanthus sucuuba*, a Central Amazon tree. Aquat. Bot. 90, 246–252

[B19] García-Sánchez F. Syvertsen J. P. Gimeno V. Botía P. Perez-Perez J. G. (2007). Response to flooding and drought stress by two citrus rootstock seedlings with different water-use efficiency. Physiol. Plant. 130, 532–542

[B20] Geigenberger P. (2003). Response of plant metabolism to too little oxygen. Environ. Pollut. 116, 31–35 12753974 10.1016/s1369-5266(03)00038-4

[B21] Graffmann K. Grosse W. Junk W. J. Parolin P. (2005). Pressurized gas transport in Amazonian floodplain trees. Biotropica 37, 609–619

[B22] Grosse W. Frye J. Lattermann S. (1992). Root aeration in wetland trees by pressurized gas transport. Tree Physiol. 10, 285–295 10.1093/treephys/10.3.285 14969985

[B23] Herrera A. Escala M. Rengifo E. (2009). Leaf anatomy changes related to physiological adaptations to flooding in Amazonian tree species. Brazil. J. Plant Physiol. 21, 301–308

[B24] Herrera A. Rengifo E. Tezara W. (2001). Leaf gas exchange of trees in a tropical seasonally flooded forest, in The Tree 2000, ed Labrecque M. (Montreal, QC: Isabelle Quentin), 257–262

[B25] Herrera A. Tezara W. Rengifo E. Flores S. (2008a). Changes with seasonal flooding in sap flow of the tropical flood-tolerant tree species, *Campsiandra laurifolia*. Trees 22, 551–558

[B26] Herrera A. Tezara W. Marín O. Rengifo E. (2008b). Stomatal and non-stomatal limitations of photosynthesis in trees of a tropical seasonally flooded forest. Physiol. Plant. 134, 41–48 10.1111/j.1399-3054.2008.01099.x 18444960

[B27] Islam M. A. Macdonald S. E. (2004). Ecophysiological adaptations of black spruce (*Picea mariana*) and tamarack (*Larix laricina*) seedlings to flood. Trees 18, 35–42

[B28] Izquierdo L. (1988). Efecto de la Inundación Sobre la Actividad Metabólica en Raíces de una Especie Arbórea, Acosmium nitens (Vog.) Yakoul (Papilonaceae) de áreas Estacionalmente Inundables. Licenciado thesis, Universidad Central de Venezuela, Caracas.

[B29] Jackson M. (1997). Hormones from roots as signals for the shoots of stressed plants. Trends Plant Sci. 2, 22–28

[B30] Jackson M. B. Colmer T. D. (2005). Response and adaptation by plants to flooding stress. Ann. Bot. 96, 501–505 10.1093/aob/mci205 16217870 PMC4247020

[B31] James E. K. Crawford R. M. M. (1998). Effect of oxygen availability on nitrogen fixation by two *Lotus* species under flooded conditions. J. Exp. Bot. 49, 599–609

[B32] Joly C. A. Crawford R. M. M. (1982). Variation in tolerance and metabolic responses to flooding in some tropical trees. J. Exp. Bot. 33, 799–809

[B33] Konnerup D. Sorrell B. K. Brix H. (2011). Do tropical wetland plants possess convective gas flow mechanisms? New Phytol. 190, 379–386 10.1111/j.1469-8137.2010.03585.x 21175639

[B34] Kozlowski T. T. (1984). Plant responses to flooding of soil. BioScience 34, 162–167

[B35] Kozlowski T. T. (1997). Responses of woody plants to flooding and salinity. Tree Physiol. Monogr. 1, 1–29

[B36] Kreuzwieser J. Furniss S. Rennenberg H. (2002). Impact of waterlogging on the N-metabolism of flood tolerant and non-tolerant tree species. Plant Cell Environ. 25, 1039–1049

[B37] Kreuzwieser J. Papadopoulou E. Rennenberg H. (2004). Interaction of flooding with carbon metabolism of forest trees. Plant Biol. 6, 299–306 10.1055/s-2004-817882 15143438

[B38] Lopez O. R. Kursar T. A. (1999). Flood tolerance of four tropical tree species. Tree Physiol. 19, 925–932 10.1093/treephys/19.14.925 12651304

[B39] McElrone A. J. Bichler J. Pockman W. T. Addington R. N. Linder C. R. Jackson R. B. (2007). Aquaporin-mediated changes in hydraulic conductivity of deep tree roots accessed via caves. Plant Cell Environ. 30, 1411–1421 10.1111/j.1365-3040.2007.01714.x 17897411

[B40] Melack J. M. Hess L. L. Gastil M. Forsberg B. R. Hamilton S. K. I. Lima B. T. (2004). Regionalization of methane emissions in the Amazon Basin with microwave remote sensing. Glob. Change Biol. 10, 530–544

[B41] Mielke M. S. de Almeida A.-A. F. Gomes F. P. Aguilar M. A. G. Mangabeira P. A. O. (2003). Leaf gas exchange, chlorophyll fluorescence and growth responses of *Genipa americana* seedlings to soil flooding. Environ. Exp. Bot. 50, 221–231

[B42] Mommer L. Pons T. Wolters-Arts M. Venema J. Visser E. (2005). Submergence-induced morphological, anatomical, and biochemical responses in a terrestrial species affect gas diffusion resistance and photosynthetic performance. Plant Physiol. 139, 497–508 10.1104/pp.105.064725 16126859 PMC1203398

[B43] Otte M. L. (2001). What is stress to a wetland plant? Environ. Exp. Bot. 46, 195–202

[B44] Parolin P. (2000). Phenology and CO_2_-assimilation of trees in Central Amazonian floodplains. J. Trop. Ecol. 16, 465–473

[B45] Parolin P. (2001). Morphological and physiological adjustments to waterlogging and drought in seedlings of Amazonian floodplain trees. Oecologia 128, 326–335 10.1007/s004420100660 24549901

[B46] Parolin P. Armbrüster N. Junk W. J. (2002). Seasonal changes of leaf nitrogen content in trees of amazonian floodplains. Acta Amazon. 32, 123–132

[B47] Parolin P. Armbrüster N. Junk W. J. (2006). Two Amazonian flooplain trees react differently to periodical flooding. Trop. Ecol. 47, 243–250

[B48] Parolin P. Wittmann F. (2010). Struggle in the flood: tree responses to flooding stress in four tropical floodplain systems. AoB Plants 2010:plq003 10.1093/aobpla/plq003 22476061 PMC2965040

[B49] Perata P. Armstrong W. Voesenek L. A. C. J. (2011). Plants and flooding stress. New Phytol. 190, 269–273 10.1111/j.1469-8137.2011.03702.x 21443603

[B50] Pezeshki S. R. (1987). Gas exchange response of tupelo-gum (*Nissa aquatica* L.) to flooding and salinity. Photosynthetica 21, 489–493

[B51] Pezeshki S. R. (1993). Differences in patterns of photosynthetic responses to hypoxia in flood-tolerant and flood-sensitive tree species. Photosyntetica 28, 423–430 14871732

[B52] Prance G. T. (1979). Notes on the vegetation of Amazonia. III. Terminology of Amazonian forest types subjected to inundation. Brittonia 31, 26–38

[B53] Rengifo E. Herrera A. Tezara W. Flores S. (2006). Efecto de la Inundación y la Sequía Sobre la Conductancia Estomática, el Contenido ABA Xilemático y Foliar y el Estado Hídrico de dos Especies Arbóreas Tropicales Tolerantes a la Inundación. Santo Domingo: IX Congreso Latinoamericano de Botánica

[B54] Rengifo E. Tezara W. Herrera A. (2005). Water relations, chlorophyll a fluorescence and carbohydrate contents in trees of a tropical forest in response to flood. Photosynthetica 43, 203–210

[B55] Roças G. Scarano F. R. (2001). Leaf anatomical variation in *Alchornea triplinervia* (Spreng) Müll. Arg. (Euphorbiaceae) under distinct light and soil water regimes. Trop. Ecol. 47, 243–250

[B56] Rosales J. (1988). Análisis Florístico-Estructural y Algunas Relaciones Ecológicas en un Bosque Inundable en la Boca del río Mapire, Edo. Anzoátegui. M.Sc. thesis, Instituto Venezolano de Investigaciones Científicas, Caracas, 233.

[B57] Roth I. Lindorf H. (1991). Leaf structure of species of *Pachira* indigenous of Venezuela from different habitats. Bot. Jahrb. Syst. 113, 203–219

[B58] Ruiz-Sánchez M. C. Domingo R. Morales D. Torrecillas A. (1996). Water relations of Fino lemon plants on two rootstocks under flooded conditions. Plant Sci. 120, 119–125

[B59] Snyder K. A. James J. J. Richards J. H. Donovan L. A. (2008). Does hydraulic lift or nighttime transpiration facilitate nitrogen acquisition? Plant Soil 306, 159–166

[B60] Sun O. J. Sweet G. B. Whitehead D. Buchan G. D. (1995). Physiological responses to water stress and waterlogging in *Nothofagus* species. Tree Physiol. 15, 629–638 10.1093/treephys/15.10.629 14965996

[B61] Tournaire-Roux C. Sutka M. Javot H. Gout E. Gerbeau P. Luu D.-T. (2003). Cytosolic pH regulates root water transport during anoxic stress through gating of aquaporins. Nature 425, 393–397 10.1038/nature01853 14508488

[B62] Vegas-Vilarrúbia T. Herrera R. (1993a). Seasonal alternation of lentic/lotic conditions in the Mapire system, a tropical floodplain lake in Venezuela. Hydrobiologia 262, 43–55

[B63] Vegas-Vilarrúbia T. Herrera R. (1993b). Effects of periodic flooding on the water chemistry and primary production of the Mapire system (Venezuela). Hydrobiologia 262, 31–42

[B64] Waldhoff D. (2009). Leaf structure in trees of Central Amazonian floodplain forests (Brazil). Environ. Exp. Bot. 66, 135–142

[B65] Waldhoff D. Furch B. (2002). Leaf morphology and anatomy in eleven tree species from Central Amazonian floodplains (Brazil). Amazoniana 17, 79–94

[B66] Waldhoff D. Furch B. Junk W. (2002). Fluorescence parameters, chlorophyll concentration, and anatomical features as indicators for flood adaptation of an abundant tree species in Central Amazonia: *Symmeria paniculata*. Environ. Exp. Bot. 48, 225–235

[B67] Wang W. Yan Z. You S. Zhang Y. Chen L. Lin G. (2011). Mangroves: obligate or facultative halophytes? A review. Trees 25, 953–963

